# Idiopathic Aneurysm of the Distal Radial Artery in the Fossa Tabatiére

**DOI:** 10.7759/cureus.86520

**Published:** 2025-06-22

**Authors:** Jana Pobehová, Martina Zavacká, Pavol Pobeha

**Affiliations:** 1 Clinic of Vascular Surgery, East Slovak Institute of Heart and Vascular Diseases (VÚSCH), Košice, SVK; 2 Medical Faculty, Pavol Jozef Šafárik University, Košice, SVK

**Keywords:** angiosurgery, artery ligation, fossa tabatiére, radial artery aneurysm, resection

## Abstract

The aneurysms of the radial arteries are extremely rare. Aneurysm of the radial artery is defined as a focal dilation of all components of the vascular wall to a diameter greater than 1.5 times its lumen. In our case report, we present the diagnosis, management, and angiosurgical treatment of a patient with an extremely rare case of aneurysm on the distal radial artery in the fossa Tabatiére. Angiosurgical treatment, which is always recommended due to the risk of rupture, embolization, and ischemia of the distal parts of the hand, has a low morbidity.

## Introduction

Aneurysms of the distal part and the dorsal ramus of the radial artery are extremely rare. Thus far, approximately 20 cases of true arterial bulging localized in the fossa Tabatiére have been described. This triangular-shaped area with a slight depression, located ventromedial and distal to the wrist, is anatomically bounded on the medial side by the tendon of the m. extensor pollicis brevis, laterally by the tendon of the m. extensor pollicis longus, and proximally by the extensor retinaculum musculus. The bottom is formed by the styloid process of the radius and partly by the scaphoid bone. Epifascially, in this location runs the v. cephalica and the superficial branch of the radial nerve. In depth, the radial artery and its ramus carpalis dorsalis and radial veins run. The artery runs relatively unprotected here. The name fossa la tabatière is French for "snuffbox," because tobacco was placed in it for "sniffing."

Aneurysm of the radial artery is defined as a focal dilation of all components of the vascular wall to a diameter greater than 1.5 times its lumen. The physiological diameter of the radial artery lumen is 2-3 mm [1]. False aneurysms (pseudoaneurysms) arise secondarily, most often after catheterization through the radial artery [1]. Only a certain layer of the vascular wall is dilated, most often the tunica adventitia. Arterial dilations mostly arise secondarily after injury, but mycotic, infectious, atherosclerosis-related aneurysms, connective tissue disorders, vascular tumors, and idiopathic aneurysms have also been described [2-8]. 

Early diagnosis and treatment are important to prevent potential complications: thrombus formation, distal digital ischemia, and rupture with bleeding. In our case report, we present the diagnosis, management, and vascular surgery treatment of a patient with an extremely rare case of aneurysm of the distal radial artery in the fossa Tabatiére.

## Case presentation

A 61-year-old postmenopausal patient, previously followed by an angiologist, was admitted to our department for an ultrasound-verified aneurysm of the distal part of the a. radialis on the right hand, with an extension into the area of ​​the first metacarpophalangeal joint. Her anamnestic history indicated a pulsating mass in the area between the right thumb and the inner side of the wrist, which she had observed for approximately nine months. According to her, the mass had been growing slightly. The patient did not report any trauma or intervention in the given area. There was no similar case in the family. She also reported treatment for subclinical hypothyroidism for goiter and surgery for varicose veins on both lower extremities. There was a family history of oncological diseases (ovarian cancer) and a sudden stroke. The medication she had been taking for a long time included the venotonics and substitution drugs for hypothyroidism. She reported penicillin as an allergy.

During the ultrasonography, the angiologist repeatedly described aneurysmal dilatation of the distal part of the right radial artery with an overlap on the dorsal branch of the right radial artery in diameter 10 mm and length 12 mm, partially filled with thrombi. During the physical examination of the right upper limb, we palpated a pulsating formation in the area of ​​the right fossa Tabatiére, prominent above the I. metacarpophalangeal joint. Pulsation on the a. radialis on the right above the wrist was preserved. Given the clearly visualized aneurysm and adequate flow rates above and below the site of the aneurysm and considering the risk of using other interventional imaging methods with a relatively clear diagnosis, we indicated vascular surgery. 

**Figure 1:**
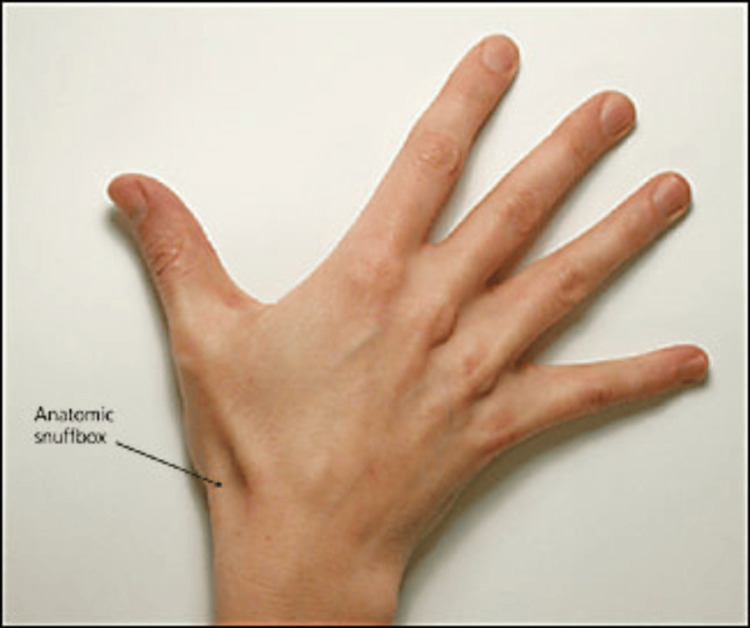
Fossa Tabatiére

**Figure 2:**
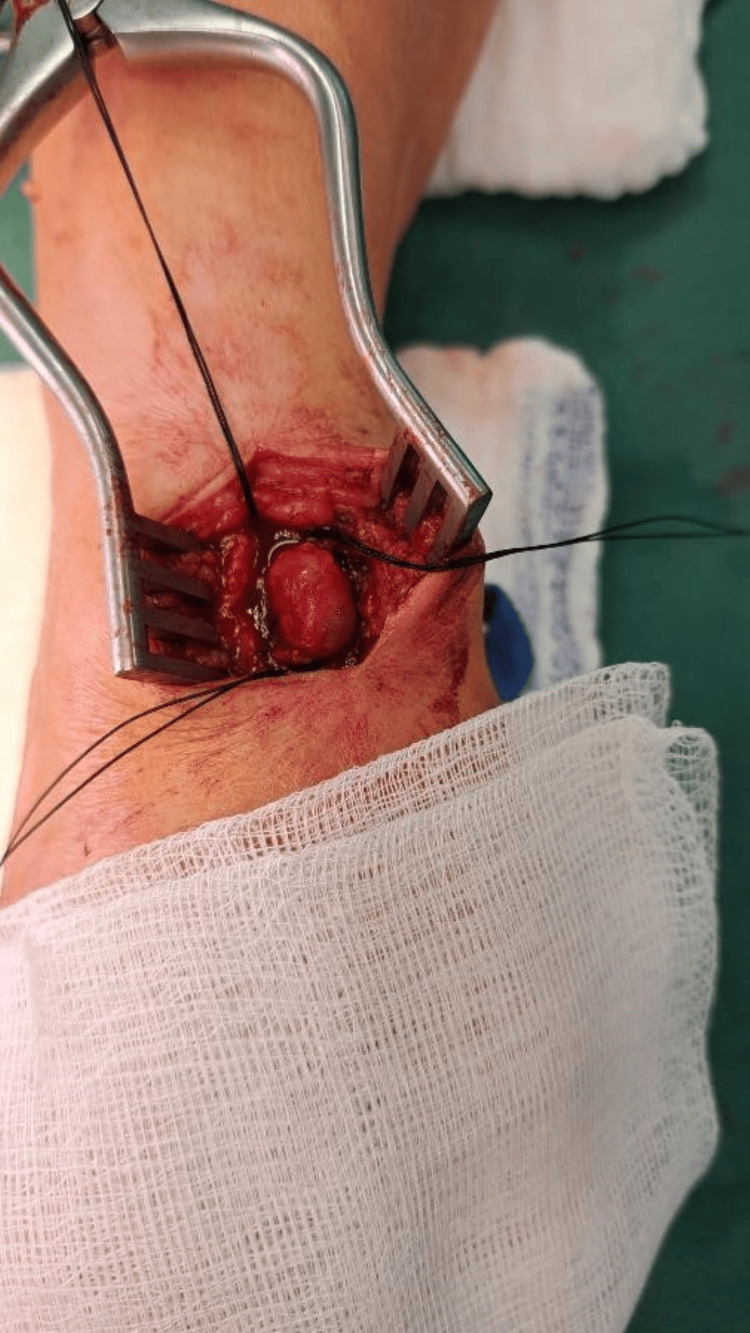
Ligation of the distal radial aneurysm (right hand Fossa Tabatiere)

**Figure 3:**
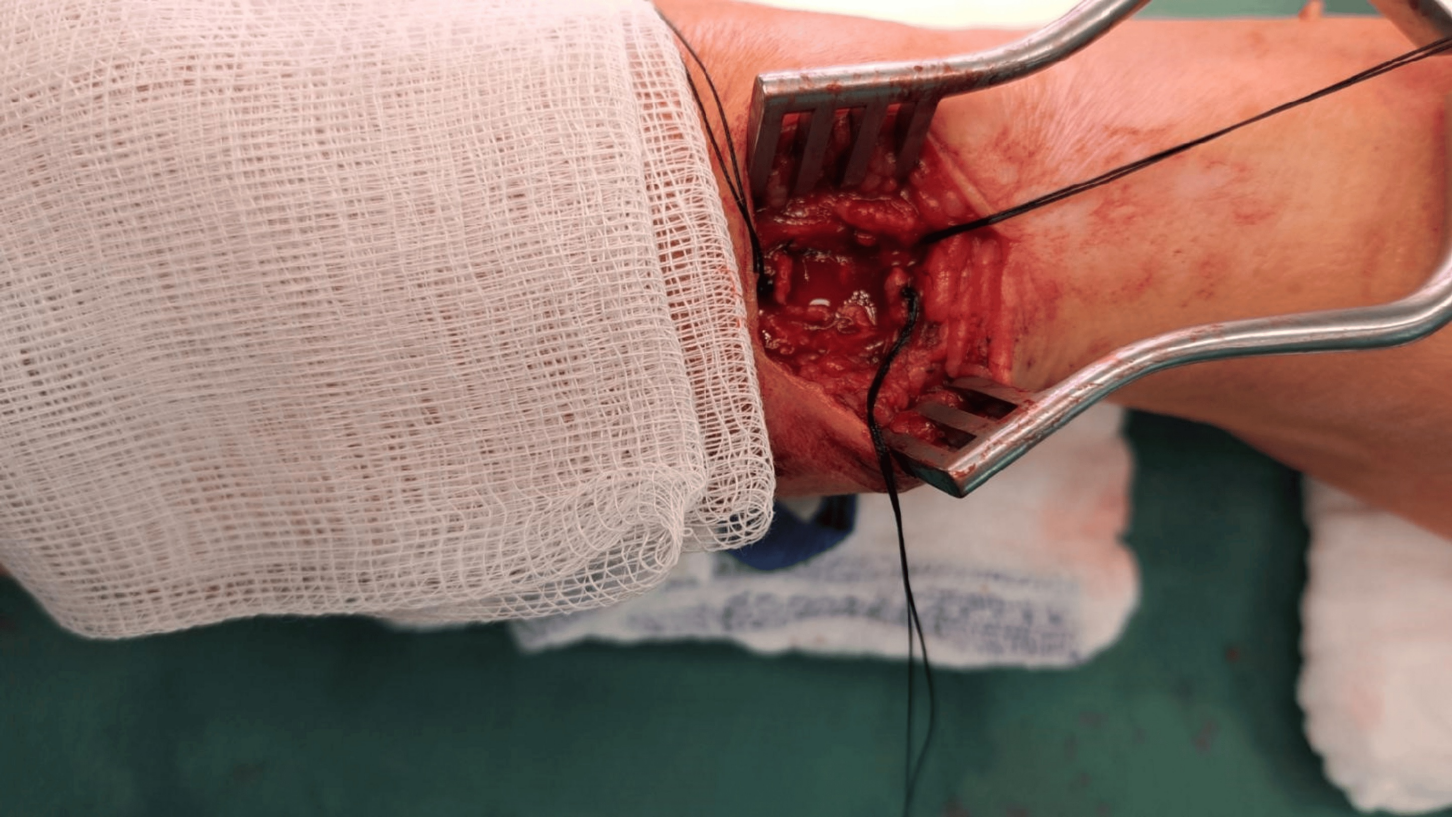
Extirpation of the distal radial aneurysm

**Figure 4:**
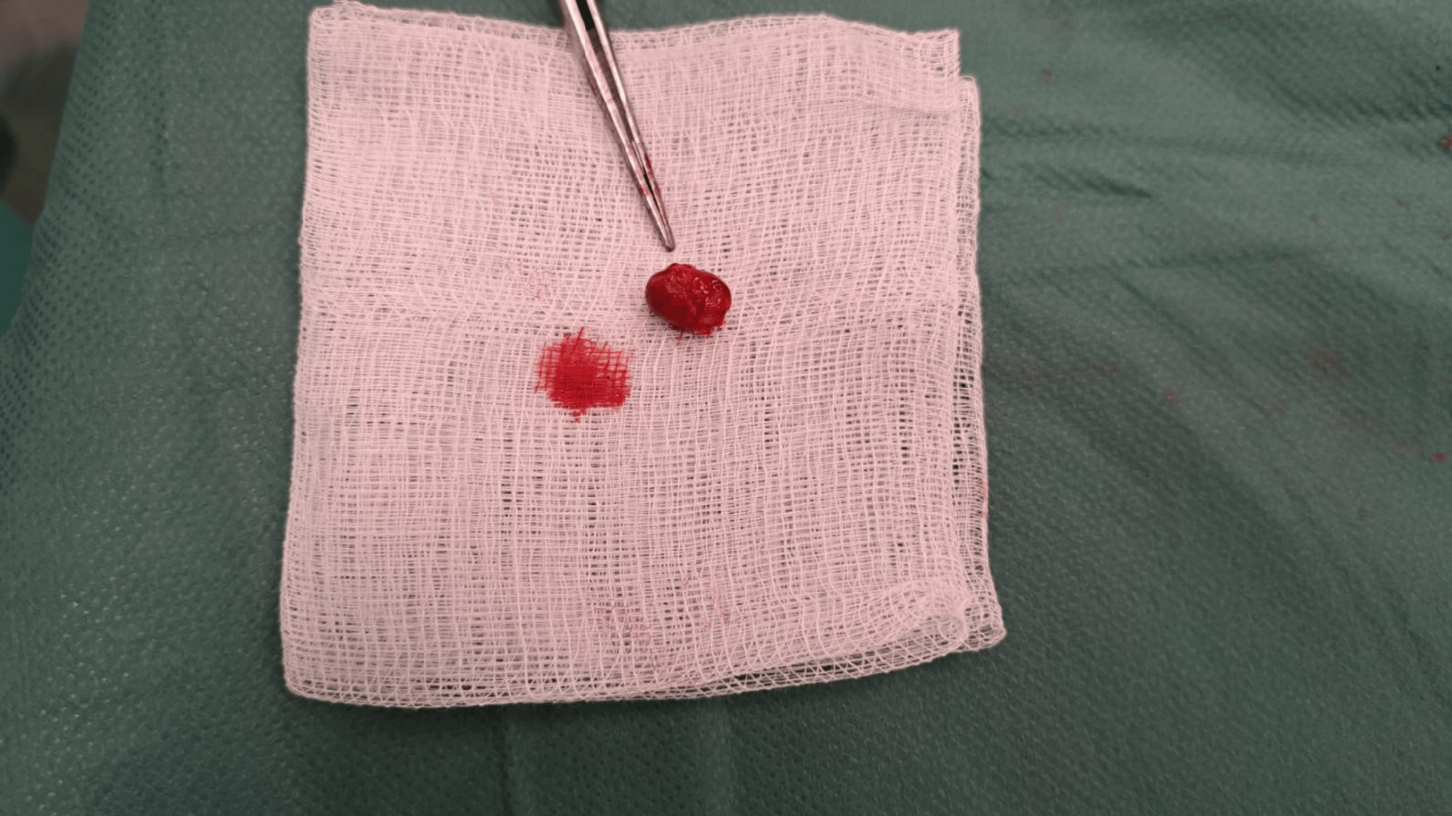
Distal radial aneurysm after extirpation

After the necessary preoperative preparation, the patient was operated on under local anesthesia. We made a longitudinal incision above the resistance and dissected the dorsal branch of the right radial artery in the Tabatiere fossa. We gradually ligated the small branches and then proceeded to ligate the aneurysmal dilation at the proximal and distal ends (Figure 1). Intraoperatively, we verified whether there were any signs of ischemia or disorders of sensitivity and movement of the thumb. Given that no symptoms occurred, we decided to resect the aneurysm of the right dorsal radial artery, preserving the main afferent artery of the right radial artery. If intraoperatively there were signs of ischemia of the right hand and thumb, reconstruction of the artery would be necessary. Since there were no signs of ischemia and it was not a magisterial artery, we decided to exclude the aneurysm (Figure 2), which was filled with both older and fresh thrombi according to the USG examination. We sent the aneurysm for histological examination (Figures 3, 4). After hemostasis in the surgical wound and irrigation, we sutured the skin with individual self-absorbable sutures and inserted a drain into the wound. 

Postoperatively, she was covered with antibiotics and a preventive dose of low-molecular-weight heparin (LMWH). On the next day, after dressing and drain extraction, she was discharged to home care. During the outpatient check-up, we extracted the sutures, the surgical wound was healed primarily, a radial artery was palpable, and the patient had no subjective or objective complaints. Histological examination verified a right arterial aneurysm in the Tabatiére fossa. We recommended regular angiological checks once a year.

## Discussion

Aneurysms of the upper extremity arteries are extremely rare. The prevalence of radial artery aneurysms is only 2.9% of all aneurysms affecting the upper extremity arteries [9]. This rarity is attributed to the fact that the radial artery has a small lumen. Based on Laplace's law, there is only a low probability that an aneurysm will form in a vessel with a small lumen. Higher arterial pressures are required for its growth and dilation [10,11]. Diagnosis of radial artery aneurysms is usually performed using noninvasive methods such as ultrasonography, computed tomography angiography (CTA), and magnetic resonance angiography (MRA) [12]. Invasive angiography (DSA) may also be performed, especially when endovascular treatment is being considered. Angiosurgical treatment, which is always recommended due to the risk of rupture, embolization, and ischemia of the distal parts of the hand, has a low morbidity [13,14].

In our case, the patient complained of a pulsatile mass in the area above the right thumb, without the presence of pain or paresthesia. The diagnosis of the aneurysm was made clinically with the findings of physical examination of the pulsatile mass, together with ultrasonography, which visualized the dimensions and flow through the aneurysm and verified normal anatomical proportions above and below the bulge. The aneurysm was excised and ligated in the patient. The diagnosis was later confirmed by intraoperative examination and histological examination.

Of the cases worldwide and published so far (Table 1), 13 aneurysms of the radial artery in the Tabatiére fossa have been described: eight in men and five in women, with a mean age of 59.6 years. The aneurysms ranged in size from 11 to 77 mm. The vast majority were idiopathic, one was post-traumatic, and one was due to a connective tissue disease (Marfan syndrome). Almost all were primarily diagnosed using USG; in most cases, CTAG or angiography was added. Only two groups of authors left the aneurysm (15 mm in size) under observation, without surgical intervention [15]. Half of the authors of the published works chose vascular surgery excision and ligation of the distal part of the radial artery; in two cases, a part of the artery was replaced by the great saphenous vein and cephalic vein. The rest of the cases were reconstructed primarily by end-to-end or end-to-side anastomosis. The possibilities of arterial reconstruction are various. They depend on the local findings, sufficient vascular supply of the hand (the limb must not be endangered by ligation of the aneurysmally dilated artery), patency of the palmar arch, arterial supply of the ulnar artery, a suitable autologous vein, and skills and experience of the vascular surgeon. To evaluate the dominance of the radial or ulnar artery, there are various tests with different sensitivities and specificities, including the Allen test, the modified Allen test, digital plethysmography, digital Doppler curves, pressures, and duplex ultrasonography. In the differential diagnosis, it is necessary to distinguish a possible ganglion, neuroma, lipoma, and synovial cyst. Incorrect diagnosis is associated with higher morbidity. 

**Table 1:** Cases of aneurysms of the radial artery in the Tabatiére fossa published in the literature USG: ultrasonography, CTAG: cardiotocography

Vascular surgery/author	Localization/diameter/mm/	Etiology	Sex/age	Diagnostics
Excision, primary end-to-side anastomosis/Thorrens et al. (1966)	Snuffbox/30	Idiopathic	M/60	Angiography
Excision and primary anastomosis/Malt et al. (1978)	Snuffbox/20	Idiopathic	M/56	Angiography
Excision and primary end-to-end anastomosis/Al-Zoubi et al. (2018)	Wrist/30	Idiopathic	M/61	USG, CTAG
Excision+ligation (right), observation (left)/Yukios et al. (2009)	Snuffbox/ 9 (right) 5 (left)	Marfanś syndrome	F/74	USG
Excision and repair with interposition of VSM graft/Ghaffarian et al. (2018)	Snuffbox/6,3	Idiopathic	M/25	USG, CTAG
Excision, artery ligation/Luzzani et al. (2006)	Snuffbox/11	Idiopathic	F/63	USG, MRA, angiography
Excision and artery ligation/Jedynak et al. (2012)	Snuffbox/not stated	Idiopathic	M/60	USG, CTAG
Excision and artery ligation/Igari et al. (2013)	Snuffbox/15	Idiopathic	F/72	Not stated
Excision and artery ligation/Shaabi et al. (2014)	Snuffbox/20	Idiopathic	F/65	CTAG
Excision and artery ligation/Maalouly et al. (2019)	Snuffbox/15	Idiopathic	F/73	CTAG
Excision and artery ligation	Snuffbox, at the base of the thumb/19	Repetitive occupational injury	M/62	USG
Excision and artery ligation/Gabriel et al. (2013)	Wrist/18,8	Idiopathic	M/49	USG
Excision and artery ligation/DeSer et al. (2017)	Wrist/20	Bechcetś disease	M/25	USG
Excision, primary anastomosis of the radial artery to the second digital artery, first digital artery ligated/Filis et al. (2007)	Wrist/30	Idiopathic	M/45	Angiography

The options for vascular surgery treatment include the following: 1) from the simplest resection and ligation of the radial stump, if the hand is adequately perfusion; 2) reconstruction with primary end-to-end anastomosis, if there is no tension in the reconstructed part of the artery; and 3) graft interposition in the case of a defect, which is more lengthy and requires the presence of a sufficient autologous vein (great saphenous vein and cephalic vein) [1].

There is no clear consensus on whether it is sufficient to simply ligate or reconstruct the artery [1]. Some authors recommend revascularization whenever possible; other authors argue that selective revascularization depends on collateral circulation [10,11]. Both methods achieve good results with low morbidity.

## Conclusions

Idiopathic aneurysms of the radial artery in the Tabatiére fossa are rare. Currently, there are no completely precise guidelines regarding the risk of embolization and rupture. Vascular surgery treatment is almost always recommended due to the risk of possible complications, with minimal morbidity, which is finally demonstrated by the case described by us and other foreign publications dealing with this issue.
